# Potential Role of Induced Pluripotent Stem Cells (IPSCs) for Cell-Based Therapy of the Ocular Surface

**DOI:** 10.3390/jcm4020318

**Published:** 2015-02-12

**Authors:** Ricardo P. Casaroli-Marano, Núria Nieto-Nicolau, Eva M. Martínez-Conesa, Michael Edel, Ana B. Álvarez-Palomo

**Affiliations:** 1 Department of Surgery, School of Medicine and Hospital Clínic de Barcelona (IDIBAPS), University of Barcelona, Calle Sabino de Arana 1 (2nd floor), E-08028 Barcelona, Spain; 2 CellTec-UB and the Clinic Foundation for Biomedical Research (FCRB), University of Barcelona, Avda. Diagonal 643, E-08028 Barcelona, Spain; E-Mail: nurianieto@ub.edu; 3 Tissue Bank of BST (GenCat), Calle Dr Antoni Pujadas 42, SSMM Sant Joan de Déu, Edifici Pujadas, E-08830 Sant Boi de Llobregat, Spain; E-Mail: emmartinez@bst.cat; 4 Pluripotency Group, Department of Physiology I, School of Medicine, University of Barcelona, Calle Casanovas 143, E-08036 Barcelona, Spain; E-Mails: edel-michael@gmail.com (M.E.); b.alvarez@ub.edu (A.B.A.-P.); 5 Faculty of Medicine, Children’s Hospital, University of Sydney, 2006 NSW, Australia

**Keywords:** limbal stem cells, cornea, limbal stem cell deficiency, epithelial differentiation, human adult progenitor cells, *ex vivo* expansion, cell culture, ocular burns, cell-based therapy, human stem cells

## Abstract

The integrity and normal function of the corneal epithelium are crucial for maintaining the cornea’s transparency and vision. The existence of a cell population with progenitor characteristics in the limbus maintains a dynamic of constant epithelial repair and renewal. Currently, cell-based therapies for bio replacement—cultured limbal epithelial transplantation (CLET) and cultured oral mucosal epithelial transplantation (COMET)—present very encouraging clinical results for treating limbal stem cell deficiency (LSCD) and restoring vision. Another emerging therapeutic approach consists of obtaining and implementing human progenitor cells of different origins in association with tissue engineering methods. The development of cell-based therapies using stem cells, such as human adult mesenchymal or induced pluripotent stem cells (IPSCs), represent a significant breakthrough in the treatment of certain eye diseases, offering a more rational, less invasive, and better physiological treatment option in regenerative medicine for the ocular surface. This review will focus on the main concepts of cell-based therapies for the ocular surface and the future use of IPSCs to treat LSCD.

## 1. Introduction

**Figure 1:**
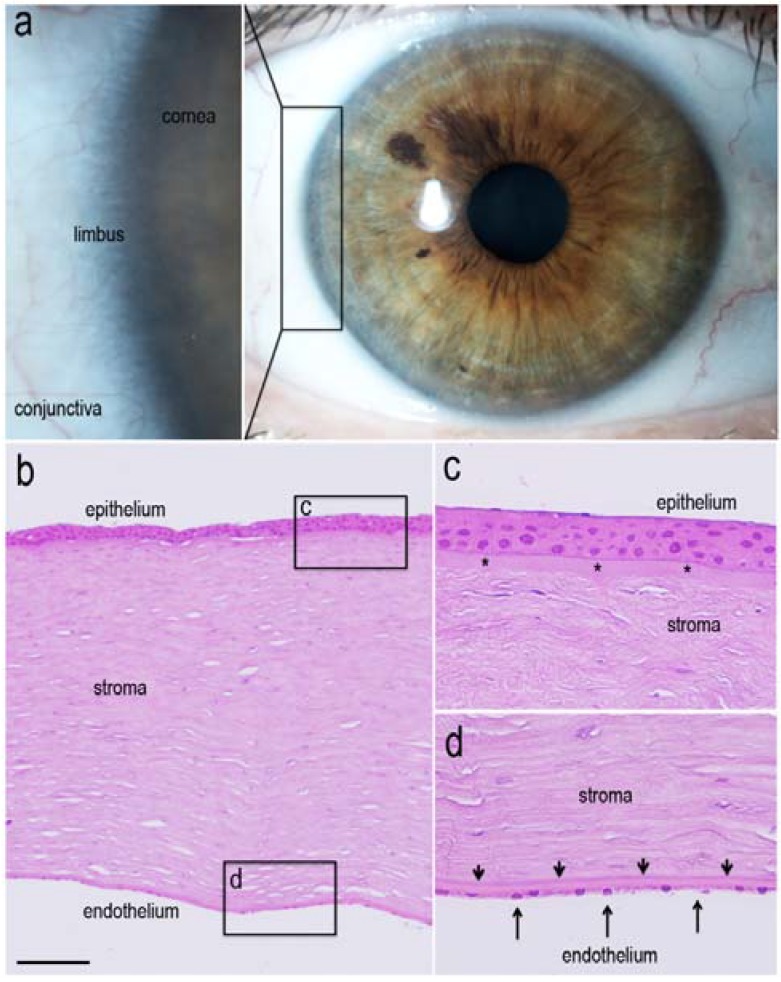
The corneal limbus is the circumferential anatomic area, approximately 1.5 mm wide, which separates the clear cornea from the opaque sclera (**a**); The limbal region represents the “reservoir” for LSCs in the ocular surface. In a cross-section of the human cornea stained with hematoxylin-eosin, (**b**) to (**d**), details of its main layers can be observed. The cornea is composed of a stratified non-keratinized squamous epithelial layer (epithelium), the stroma and an endothelial cuboidal layer (endothelium) (**b**); The corneal epithelium (48 to 55 µm thick) consists of the outermost layer, which presents five to seven stratified cell layers (**c**), limited posteriorly by Bowman’s layer (10 to 12 µm thick; c, asterisk). The stroma (480 to 510 µm thick; b), composed of compacted collagen lamellae and keratocytes (c and d), offers transparency and scaffolding to maintain the shape of the cornea in its middle portion. The stroma is separated from the endothelium (about 5 µm thick; d, large arrows) by Descemet’s membrane (8 to 10 µm thick; d, narrow arrows), which acts as a basement membrane for the corneal endothelial cells (**d**). Bar = 150 µm for b; Bar = 25 µm for c and d.

The ocular surface is mainly composed of the cornea and the conjunctiva with their epithelia. The cornea is the primary refractive element at the anterior surface of the eye that is responsible for approximately two-thirds of its total optical power. Basically, the cornea is composed of five well-defined layers (Figure 1). It consists of an outermost stratified, squamous and non-keratinized epithelial layer (corneal epithelium) limited posteriorly by Bowman’s layer. The underlying stroma, which accounts for about 90% of the middle thickness of the cornea, comprises aligned arrays of collagen fibrils interspersed with cellular components (keratocytes) and it is this highly organized arrangement of lamellae that is responsible for the cornea’s transparency. The stroma is separated from the endothelial layer (corneal endothelium) by Descemet’s membrane, which acts as a basement membrane for these endothelial cells. The corneal endothelium is a single cuboidal layer of metabolically active cells that are in direct contact with the aqueous humor in the anterior chamber. These cells help to maintain corneal transparency by actively pumping water out of the stroma [1]. The corneal epithelium has a key role in keeping the cornea transparent and free of blood vessels and, to this end, presents permanent repair phenomena essential for the conservation of the cornea’s physiology [1,2,3]. The homeostasis of the corneal epithelium is crucial to maintaining the structural integrity of the ocular surface, the transparency of the cornea and visual function.

### 1.1. Limbal Stem Cells

It has been observed that progenitor cells responsible for the continual renewal of the corneal epithelium are located in the basal layers of the sclerocorneal limbus. The human limbus—the circumferential anatomic area (approximately 1.5 mm wide) that separates the clear cornea from the opaque sclera, which is covered by conjunctiva—serves as the “reservoir” for the stem cells and also provides a barrier to the overgrowth of conjunctival epithelial cells and its blood vessels onto the cornea [1,2,3] (Figure 1). Due to their particularities, the *limbal stem cells* (LSCs) have a crucial role in maintaining the integrity and in the renewal events of corneal epithelium. Their main features are highlighted: it is their behavior as oligopotent progenitor cells, with high nuclear-cytoplasmic ratio a slow cell cycle, and a high proliferative potential that adds its great capacity for self-renewal by asymmetric division [3,4,5]. In the limbus, it is possible to identify several cell subpopulations of different progenies (typical progenitors and amplifying cells at different stages of differentiation), melanocytes, antigen-presenting and mesenchymal cells, vascular elements and nerve endings that form a specialized and unique environment called *niche*. This particular microenvironment is considered responsible for the proliferative and self-renewal cellular characteristics of the limbal region [2,3,6]. The LSC niche is an anatomically defined area that is thought to provide a variety of factors, such as physical protection, survival factors and cytokines and is deemed essential to the maintenance of the “stemness” of the stem cell population while preventing entry into differentiation [2,6]. Within the niche, LSCs maintenance and function are controlled in a particular environment by several elements, including extracellular matrix components, cell adhesion molecules, and growth and survival factors secreted by stromal fibroblasts, mesenchymal stem cells and blood capillaries [6]. To date, four limbal anatomic structures have been proposed as the corneal stem cell niche [2,6], Palisades of Vogt, limbal epithelial crypts [7], limbal crypts and focal stromal projections [8].

### 1.2. Renewal of Corneal Epithelium

It has been shown that cell subpopulations with progenitor features, located in the deeper basal layers of the corneal epithelium, have the capacity to differentiate into post-mitotic cell populations located in the outermost epithelial layers. This continuous centripetal movement (the XYZ hypothesis)—from the peripheral deeper epithelial layers to the more central outermost layers—ensures constant renewal of the corneal epithelium and maintains its integrity [1,2,3,4,5,6,9]. The *X* component represents the anterior migration from cells of the basal epithelium of the limbal region, the *Y* component represents the centripetal migration of cells from the limbus, and the *Z* component represents the desquamation from the surface of corneal epithelium. However, this XYZ theory has recently been challenged by evidence in the mouse and other mammals suggesting that uninjured cells in the central cornea can generate holoclones with characteristics of stem cells, presenting regenerative epithelial capabilities, which may also be responsible for the maintenance of the corneal epithelium [10]. Also, in support of these controversial findings, the presence of central islands of normal corneal epithelial cells has been described in patients with apparently complete clinical absence of LSCs [11]. These interesting observations may have the following interpretation: the central basal epithelial cells of the surviving corneal epithelium present the capability to regenerate, or some LSCs remain and contribute to the maintenance of the central epithelium.

### 1.3. Limbal Stem Cell Multipotency

An* in vitro* study of the clonogenic capacity of epithelial cells located in the ocular limbal region revealed a progenitor cell system stratified into levels (cellular stages or “compartments”) [2,6,12]. Undifferentiated small cells presenting progenitor cell features with high self-renewal capacity are found in the first compartment but they lose these characteristics as they migrate through the following compartments. Lastly, the final level contains a cell population with terminal differentiation features associated with little or no self-renewal capability. The latter cells, once their epithelial differentiation events are completed, lose their ability to self-renew and are incorporated as corneal epithelial cells on the surface of the central cornea. In this regard, some studies [12,13] concluded that epithelial cells of the limbal region can form holoclones with higher clonogenic potential, in contrast to epithelial cells from the central cornea. In addition, epithelial cells isolated from basal layers in the limbal region exhibit a high proliferative potential *in vitro* during expansion or in response to corneal injury [14], and show an undifferentiated phenotype lacking the expression of differentiated corneal cell markers such as cytokeratins 3 and 12 [15]. They have also been shown to retain labeled precursors of DNA for an extended time, in contrast to more differentiated cells that quickly lose them due a higher division rate [16]. This lack of differentiation and slow cell cycling are characteristics of the quiescent state of stem cells.

## 2. Limbal Stem Cell Deficiency (LSCD)

**Figure 2:**
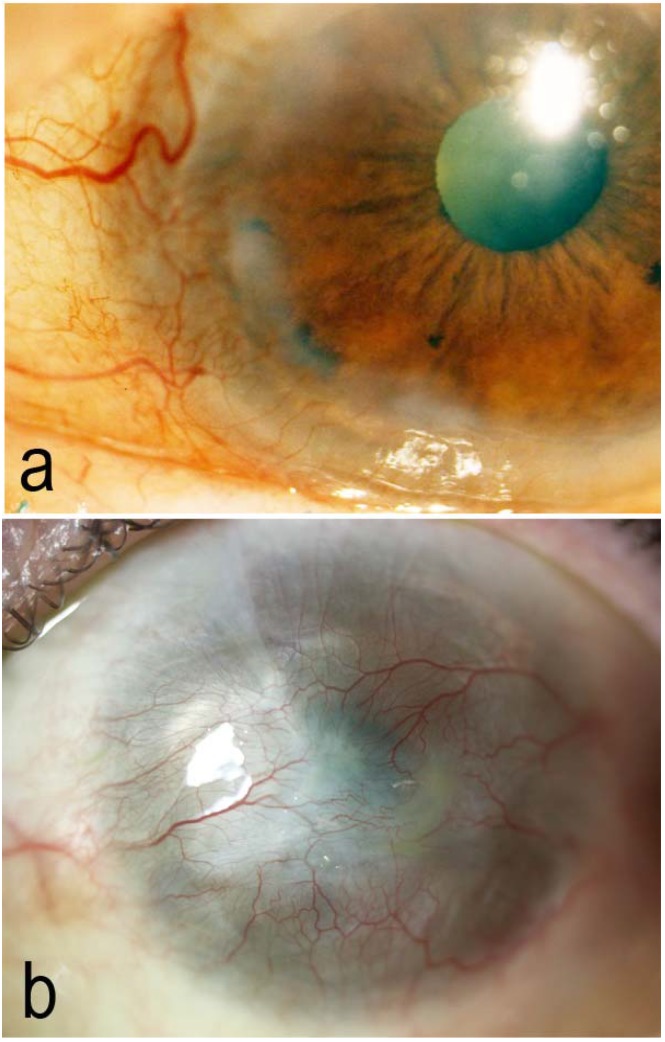
Clinical findings related to Limbal Stem Cell Deficiency (LSCD). Limbal deficiency secondary to ocular cicatricial pemphigoid with the presence of peripheral newly formed vessels leading to a loss of corneal transparency (**a**); Limbal deficiency, secondary to a chemical burn (bleach) of the ocular surface leads to a corneal conjuntivalization and neovascularization with loss of transparency (**b**). LSCD can be treated with cell therapy techniques such as cultured limbal epithelial transplantation (CLET) or cultured oral mucosal epithelial transplantation (COMET).

The disappearance, reduction or functional impairment of LSCs may produce a clinical state (limbal stem cell deficiency, LSCD) that can give rise to significant changes in the ocular surface. These changes include the occurrence of persistent corneal defects, epithelial keratinization, conjunctivalization phenomena with the development of newly formed vessels in the corneal tissue, and scarring. All this compromises the corneal physiology, reducing transparency and decreasing vision [1,2,3,4,5]. The presence of a complete loss of the corneal-limbal epithelium leads to a reactive reepithelialization by conjunctival cells, which have a high proliferative capacity. This event is followed by neovascularization, chronic inflammation with scarring of corneal stroma, causing a pronounced decrease in vision and severe discomfort (Figure 2). Furthermore, the chronic inflammatory condition not only leads to the death of more LSCs but also leaves the surviving epithelial cells unable to function properly, explaining the worsening of clinical symptoms and features over time [17,18,19,20,21,22,23,24]. In patients with severe lacrimal dysfunction syndrome (dry eye) suffering from LSCD, the conjunctival epithelium that replaced the corneal epithelium (conjunctivalization) becomes partially or totally keratinized [18,21,22,24]. Several processes and diseases (Table 1) may lead to unilateral or bilateral LSCD, and depending on its extent, the disorder can be classified as either partial or total. Chemical burns (alkalis and acids) are, however, the most frequent cause of limbal ischemia and epithelial destruction causing the loss and/or impairment of LSCs function, and are the main indication for cell-based therapy approaches [18,19,20,21,22,23,24].

**Table 1:** Main etiologies and pathological conditions for primary and secondary Limbal Stem Cell Deficiency (LSCD).

Etiology	Ocular Pathology
Idiopathic	-
Hereditary	Aniridia
	Autosomal dominant keratitis
	Gelatinous drop-like corneal dystrophy
	Iris coloboma
	Xeroderma pigmentosa
	Epidermolysis bullosa
	Dyskeratosis congenita
	Ectodermic dysplasia
	Multiple endocrine neoplasia
	Polyglandular autoimmune syndromes
Neoplasic	Intraepithelial neoplasia
	Conjuntival tumors (melanoma)
	Limbal dermoid
Degenerative	Recurrent pterygium
	Salzmann nodular corneal dystrophy
Infections	Severe infeccious keratitis
	Chlamydia conjunctivitis
Mechanical	Alkali, acid, thermal burns
	Bullous keratopathy
	Tumor excision
	Cryotherapy, radioterapy
	Systemic and local chemotherapy (MMC, 5FU)
	UV radition
	Phototherapeutic keratectomy
Anoxic	Contact lenses misuse or prolonged use
Trophic	Neurotrophic keratopathy
Inflammation	Superior limbic keratoconjunctivitis
	Collagen diseases related ulcers
	Mooren ulcer
	Atopic keratoconjunctivitis
	Ocular pemphigoid
	Ocular rosacea
	Stevens-Johnson syndrome
	Graft-versus-host disease
	Vitamin A deficiency

MMC, mitomycin-C; 5FU, 5-fluorouracil; UV, ultra-violet.

### 2.1. Cell-Based Treatments for LSCD

The concept of ocular surface reconstruction was introduced with the application of autologous conjunctiva for unilateral ocular chemical alkali burns [25]. Since then, several surgical approaches have been developed with the aim of restoring the corneal epithelium on the diseased ocular surface. In recent decades, limbal transplantation techniques using auto or allografts have been introduced as bio-replacement approaches for limbal tissues to improve and reconstruct the altered ocular surface [5]. Building on previous experience treating patients with large surface areas of burned skin, the epithelial cells of the ocular surface have been obtained by cell culture techniques for *ex vivo* expansion. Subsequently, the ocular surface was successfully reconstructed by using LSCs in patients with severe unilateral ocular surface pathology [17]. Since then, various translational approaches have been developed and optimized, with satisfactory long-term clinical results [18,19,20,21,22,23].

Treatment approaches for LSCD can be divided into three main categories [4,5,23,24]: (a) transplants and bio-replacement of tissue; (b) cell-based therapy by *ex vivo* cell culture expansion; and (c) symptomatic and alternative treatment: keratoprosthesis implantation, provisional debridement of conjunctival corneal tissue, therapeutic contact lenses and drug (steroids, anti-angiogenic drugs, tear substitutes, autologous serum) therapy [5].

*Ex vivo* expansion of LSCs is the most innovative approach for ocular surface bio-replacement (CLET: cultured limbal epithelial transplantation). From a minimally invasive biopsy (1–2 mm^2^) of the healthy limbal region (the same or the contralateral eye), an explant culture technique can be applied on a suitable substrate (such as the amniotic membrane) or by separating the epithelial layer from the fragment obtained by enzymatic treatment [19,21,22,26,27,28,29]. In the latter approach, the cells obtained are *in vitro* co-cultured on feeder-layers (3T3 murine fibroblasts growth arrested by irradiation or mitomycin-C). Once cell growth is achieved, the cell suspensions are transferred to suitable substrates, such as fibrin, collagen or biocompatible polymers. The bio-replacement is carried out after removal of most of the diseased tissue from the ocular surface [17,18,19,20,21]. This methodology has many advantages over the tissue transplantation techniques used to date: essentially, it requires a substantially smaller limbal biopsy, which reduces the risk of limbal deficiency in healthy donor tissue. Its other advantages include a final high cell population that is more efficiently selected, homogeneous and, theoretically, more enriched with progenitor characteristic cells [26,27,28,29]. However, enzymatic techniques involve a more complex approach, with additional manipulation of the tissue and the need for xenoproducts at different stages of cell culture production. For its part, the explant technique has certain advantages—among them its technical simplicity, the lack of xenoproducts and its cost-effectiveness, despite the heterogeneity of the cell population cultured (sclera fibroblasts, antigen presenting cells, melanocytes, conjunctival epithelium cells and others) [21,22,26,27,28,29]. It is always desirable to use autologous cells for *ex vivo* expansion to avoid the risk of immune response. However, in the presence of severe bilateral ocular pathology, the use of heterologous epithelial cells (from cadaveric or related living donor corneas) is acceptable [18,19,20,21]. Autologous oral mucosal epithelia expanded *ex vivo* have also been successfully used as an alternative source of epithelial cells (COMET: cultured oral mucosal epithelial transplantation) [30,31].

The mechanism by which cultured LSCs may restore the ocular surface is still poorly understood. Cells may replace the progenitor population, and/or “reactivate” nonfunctioning host progenitor cells by providing stimuli for growth, or change niche behavior. It has been speculated that there may be “dormant” stem cells despite clinical features of LSCD [11].

Currently, *ex vivo* expansion methods applied in cell-based therapy for clinical application are based mostly on the use of xenogeneic or allogeneic products such as murine cells for feeder layer approaches, fetal calf or bovine serum in culture media, supplements of non-human origin for cell growth and maintenance and the human amniotic membrane as a cell carrier. These products potentially carry a risk for transmitting diseases; they may induce tumorigenesis or precipitate an immunological response in the host [18,20,21,22,23,28,29]. They also show idiosyncratic biological variability that may adversely affect the quality of cultured grafts and also the final results after transplantation. Thus, there is currently a special need to investigate options for replacing potentially hazardous xenobiotic materials with others of human origin or xeno-free chemically defined media.

### 2.2. Alternative Cell Sources for LSCD Treatment

Corneal transplantation (penetrating keratoplasty) is considered the conventional therapy to restore the corneal tissue. However, this technique is not a viable strategy for patients suffering LSCD because it does not replace the LSCs population [32]. Cell-based therapy is the most rational approach for ocular surface bio-replacement, and the ideal cells for corneal reconstruction are autologous corneal LSCs using CLET approach. Minimally invasive biopsy of the limbal tissue from the same patient’s healthy eye (unilateral disease) is the preferred method, although this source of progenitor cells is not always available. If both eyes present serious surface damage, the source of healthy LSCs will be lost; COMET is among the current therapeutic alternatives. In fact, therapy for LSCD is also rapidly evolving to include alternative cell types (of autologous or heterologous origin) and clinical approaches as treatment modalities. As a consequence, other strategies, such as the use of mesenchymal stem cells from adult tissue (bone marrow mesenchymal stromal cells or adipose derived stromal cells, among others) for cell regenerative therapy in corneal injuries, are gaining prominence at present. Other sources of cells or stem cells have been tested with regenerative aims in the ocular surface, and may be useful in situations where both eyes are affected although many of them, still without clinical use at present, but which have great translational potential (Table 2).

**Table 2:** Cell sources for *ex vivo* expansion cell-based therapy to treat Limbal Stem Cell Deficiency (LSCD).

Cell Sources	Application	References
Cultured Limbal Epithelial Cells (CLET)	Clinical application	[17,18,19,20,21]
Cultured Oral Mucosal Epithelial Cells (COMET)	Clinical application	[30,31,33,34,35,36,37]
Cultured Conjunctival Epithelial Cells	Clinical application	[38,39,40,41]
Cultured Embryonic Stem Cells	Mice model	[42,43,44,45]
Cultured Adult Epidermal Stem Cells	Goat model	[46,47,48]
Cultured Bone-Marrow Derived Mesenchymal Stem Cells	Rat and rabbit models	[49,50,51,52,53]
Cultured Adipose Derived Mesenchymal Stem Cells	*In vitro* model	[54,55]
Cultured Orbital Fat Mesenchymal Progenitor Cells	Mice model; *in vitro* model	[56,57,58]
Cultured Immature Dental Pulp Stem Cells	Rabbit model	[59,60]
Cultured Hair Follicle-Derived Stem Cells	Mice model	[61,62]
Cultured Umbilical Cord Stem Cells	Rabbit model	[63,64]

Nevertheless, the application of CLET using human amniotic membrane (hAM) or fibrin gel as a scaffold has been clinically validated and today is the most frequently used cell-based therapy applied at clinical level in ophthalmology [18,19,20,21]. Since its introduction [17], it has been used with long-term clinical follow-up periods. Despite many differences between studies regarding inclusion/exclusion criteria, the culture methods applied, transplantation techniques, and clinical outcome measures, the overall success rate of this procedure is around 70% [18,21,23]. On the other hand, oral mucosa has also been shown to be an attractive autologous epithelial cell source for cases of severe bilateral LSCD. COMET has already been used in clinical settings, offering promising long-term results with improved vision in over half of treated patients [30,31,32,33,34,35]. However, peripheral corneal neovascularization is commonly found with this approach since oral mucosal cells have greater angiogenic potential than limbal epithelial cells [65,66]. It has been suggested that these new-formed vessels may regress following local anti-angiogenic therapy [33,34,35]. Further studies are needed in this regard to assess the long-term efficacy of COMET technique. In the past five years, several clinical trials have been conducted to test, compare or consolidate the application of other approaches and other sources of progenitor cells for the treatment of LSCD (Table 3).

**Table 3:** Current cell-based therapy clinical trials for the treatment of Limbal Stem Cell Deficiency (LSCD).

Clinical Trial	Identifier	Phase	Study Characteristics	Cell Source	Situation
Corneal Epithelium Repair and Therapy Using Autologous Limbal Stem Cell Transplantation.	NCT02148016	Phase 1, Phase 2	Open label, Interventional Non-randomized, SGA	Autologous LSCs	Currently recruiting
Multicenter Study of CAOMECS Transplantation to Patients With Total Limbal Stem Cell Deficiency.	NCT01489501	Phase 3	Open label, Interventional Non-randomized, SGA	Autologous OMC	Not yet open
The Improvement of Limbal Epithelial Culture Technique by Using Collagenase to Isolate Limbal Stem Cells.	NCT02202642	Phase 1	Open label, Interventional Non-randomized, SGA	Autologous LSCs	Currently recruiting
Autologous Transplantation of Cultivated Limbal Stem Cells on Amniotic Membrane in Limbal Stem Cell Deficiency (LSD) Patients.	NCT00736307	Phase 1, Phase 2	Open label, Interventional Non-randomized, SGA	Autologous LSCs	Completed
Clinical Trial on the Effect of Autologous Oral Mucosal Epithelial Sheet Transplantation.	NCT02149732	Phase 1, Phase 2	Open label, Interventional Non-randomized, SGA	Autologous OMEC	Currently recruiting
Cultivated Stem Cell Transplantation for the Treatment of Limbal Stem Cell Deficiency (LECT).	NCT00845117	Phase 1, Phase 2	Open label, Interventional Non-randomized, SGA	Autologous LSCs	Ongoing, but not recruiting
Limbal Epithelial Stem Cell Transplantation: a Phase II Multicenter Trial (MLEC)	NCT02318485	Phase 2	Open label, Interventional Non-randomized, SGA	Allogenic or autologous LSCs	Not yet open
Cell Therapy in Failure Syndromes in Limbal Stem Cells (TC181).	NCT01619189	Phase 2	Single blind, Interventional Non-randomized, SGA	Allogenic or autologous LSCs	Currently recruiting
Autologous Cultured Corneal Epithelium (CECA) for the Treatment of Limbal Stem Cell Deficiency.	NCT01756365	Phase 1, Phase 2	Open label, Interventional Non-randomized, SGA	Autologous cultured corneal epithelium	Enrolling by invitation
Ocular Surface Reconstruction With Cultivated Autologus Mucosal Epithelial Transplantation.	NCT01942421	Phase 2, Phase 3	Open label, Interventional Non-randomized, SGA	Autologous OMEC	Ongoing, but not recruiting
Efficacy of Cultivated Corneal Epithelial Stem Cell for Ocular Surface Reconstruction.	NCT01237600	Phase 2, Phase 3	Open label, Interventional Non-randomized, SGA	Allogenic or autologous LSCs	Completed
Safety Study of Stem Cell Transplant to Treat Limbus Insufficiency Syndrome.	NCT01562002	Phase 1, Phase 2	Double blind, Interventional Randomized, Parallel assignment	Allogenic LSCs* vs.* BM-MSCs	Ongoing, but not recruiting
The Application of Oral Mucosal Epithelial Cell Sheets Cultivated on Amino Membrane in Patients Suffering From Corneal Stem Cell Insufficiency or Symblepharon.	NCT00491959	Phase 1	Open label, Interventional Non-randomized, SGA	Autologous OMEC	Completed
Transplantation of Cultivated Corneal Epithelial Sheet in Patients With Ocular Surface Disease (CLET).	NCT01123044	Phase 3	Open label, Interventional Randomized, Parallel assignment	Autologous LSCs	Unknown
Application of Cell Therapy for Ocular Surface Repair Using Progenitor Cells of Sclerocorneal Limbus Amplified *ex vivo* (MeRSO09).	NCT01470573	Phase 2	Open label, Interventional Non-randomized, SGA	Autologous LSCs	Completed
The Application of Cultured Cornea Stem Cells in Patients Suffering From Corneal Stem Cell Insufficiency.	NCT01377311	Phase 1	Open label, Interventional Non-randomized, SGA	Autologous LSCs	Completed

LSCs, limbal stem cells; OMEC, oral mucosal epithelial cells; BM-MSCs, bone marrow derived mesenchymal stem cells; SGA, single group assignment [67].

## 3. IPSCs and Corneal Epithelial Differentiation

As discussed above, adult stem cells make it possible to repair and regenerate damaged epithelial tissue. In general, these cells reside in the basal layer of the epithelium, are able to self-renew continuously, and produce transient amplifying cells (TACs) that differentiate terminally after a brief period of proliferation [68,69,70,71,72]. However, there are limitations to LSCs transplantation therapies. On one hand, for unilateral LSCD, taking biopsies from the healthy eye carries along the risk of damaging the donor eye. On the other hand, for bilateral LSCD, allogenic transplantation presents the risk of immune rejection by the patient. In this sense, induced pluripotent stem cells (IPSCs) can be obtained from minimally invasive sources from the patient himself and be differentiated into LSCs, avoiding immune rejection problems and cell availability. The discovery of IPSCs has been one of the most significant advances in regenerative medicine in the last decade. Overexpression of a specific set of transcription factors (e.g., Oct4, Sox2, c-Myc, and Klf4; or Oct4, Sox2, Lin28 and Nanog) in adult differentiated cells can reprogram cell fate and IPSCs [68,69,70]. These can be differentiated into various cell types, a property that has opened up a wide range of possibilities for the investigation of cell states, the mechanisms of differentiation, pluripotency and other related cellular identities and behaviors. Contrary to embryonic stem cells, IPSCs can be created from easy access differentiated cells, such as fibroblast or keratinocytes, and allow the creation of autologous sources of different cell types for regenerative therapies or disease modeling.

More recently, direct reprogramming of cells into different states (either pluripotent or somatic) offers one of the most promising approaches in the field of regenerative medicine, with enormous potential for examining clinical and therapeutic applications in more depth [71]. The “direct reprogramming” is characterized by a process wherein mature, fully differentiated somatic cells, can be induced to other cell types without necessarily going through a pluripotent state [71]. To this end, cells can be reprogrammed by transient overexpression of transcriptional factors for a relatively short time interval. The cells in this state are called IPS-partial cells; they respond to different signal environments (e.g., growth factors, cytokines, inductors agents, *etc*.) and have the ability to direct cell fate decisions in reprogramming [71]. For corneal repair direct reprogramming would be of great advantage by not only eliminating the pluripotent stages (potentially carcinogenic) but also avoiding the lengthy production and characterization of IPSC lines. As very few cells are needed for ocular surface cell therapy, the limited expansion capacity of IPSCs is not a limiting factor as well as the production time, which would be much shorter with an easier methodology. However, there are still very limited references to the LSCs production by transdifferentiation from easily accessible adult somatic cells. Rat adult stem cells from the bulge of hair follicle were transdifferentiated into corneal epithelial-like cells by culturing with corneal limbus soluble factors and forced overexpression of the transcripton factor Pax6 [73]. More recently, Sainchanma and colleagues [74] described a method to obtain corneal epithelial-like cells from human skin-derived precursor cells—which present some multipotency markers—by culturing them with three specific growth factors: epidermal growth factor (EGF), keratinocyte growth factor (KGF) and hepatocyte growth factor (HGF) [74]. These are encouraging results to open the way for new sources of LSCs autologous supply. However further work is necessary to refine the protocol to obtain final cells that are closer to LSCs in their marker profiling and their functionality in restoring corneal epithelium should be tested.

### 3.1. Application of IPSCs for Ocular Pathology

Regarding the application of IPSCs in the field of cell therapy for ocular pathology, IPSCs have shown great promise in treating certain degenerative retinal diseases, particularly those that affect the functionality of the retinal pigment epithelium (RPE) due to its dysfunction or loss. In this context—dry age-related macular degeneration (geographic atrophy)—cell-based therapy may be a rational and effective therapeutic alternative for certain forms of retinitis pigmentosa and gyrate atrophy [75].

The use of stem cell therapy for eye diseases presents many advantages, for a variety of reasons: (a) the intraocular environment benefits from a state of immune privilege; (b) the target tissue to be treated has certain individual anatomical and functional characteristics (defined subretinal space and specialized single stratified epithelium); (c) the intraocular space is small and limited, as is required by the treatment given the low number of cells involved; and (d) the intraocular space is easily controllable by sophisticated diagnostic imaging systems in ophthalmology that allow convenient monitoring with satisfactory clinical follow up—for example, by injection of cells under the subretinal space or into the vitreous body—which permits the visualization of the therapeutic effect and possible complications. For this reason, several recent research studies are being carried out [75,76,77].

The regeneration of the ocular surface and restoration of corneal transparency following injury is one of the fields where IPSCs may also be applicable. The first attempt to obtain LSC-like cells from pluripotent cells were carried out by Notara and colleagues [78]. Using mouse ESCs treated with conditioned media from limbal fibroblasts they obtained cells with cobblestone morphology that expressed cytokeratin (CK) 12 and ΔNp63α, opening the door for the study of pluripotent cells-derived cells in the regeneration of corneal epithelium. Yu and co-researchers [79] also obtained about 13% of conversion of mouse IPSCs to corneal epithelium-like cells by co-culture of IPSCs with corneal limbal stroma in the presence of additional growth factors related to corneal development: basic fibroblast growth factor (bFGF), EGF and nerve growth factor (NGF). Moving toward human cells, Hayashi and colleagues [80] aimed to establish IPSCs derived from human LSCs and to examine the ability of both limbal-derived and human dermal fibroblast-derived IPSCs to differentiate into corneal epithelial cells. Corneal epithelial cells were then successfully induced by the stromal cell-derived inducing activity (SDIA) differentiation method, after prolonged differentiation culture (12 weeks or later) in both, limbal (with higher corneal epithelial differentiation efficiency) and fibroblastic IPSCs. This study was the first to demonstrate a strategy for corneal epithelial cell differentiation from human IPSCs, and further suggested that an epigenomic status related to DNA methylation in specific epithelium-related genes—CK3, CK12 and Pax6—was associated with the propensity of IPSCs to differentiate into corneal epithelial cells and could be used as a criteria to choose IPSCs source for LSCs differentiation However, this protocol is lengthy and the efficiency is low, as the population obtained after the differentiation protocol is mixed with other cell types, such as RPE or lens epithelium. Ljubimov’s group [81] recently successfully generated IPSCs from human primary LSCs to re-differentiate these IPSCs back into the limbal corneal epithelium, maintaining them on natural substrate that mimicked the native LSC niche, including denuded hAM and de-epithelialized corneas. This choice of parent cells represented an improvement for limbal cell differentiation by partial retention of parental epigenetic signatures in IPSCs. The authors observed that when the gene methylation patterns were compared in IPSCs to parental LSCs, limbal-derived IPSCs presented fewer unique methylation changes than fibroblast-derived IPSCs, suggesting the retention of epigenetic memory (genes promoting methylation) during reprogramming. Interestingly, limbal-derived IPSCs cultured for two weeks on hAM induced markedly higher expression of LSC markers (ABCG2, ΔNp63, CK14, CK15, CK17, *N*-cadherin, and TrkA) than fibroblast-derived IPSCs. On hAM, the methylation profiles of select limbal-derived IPSC genes became closer to the parental cells, but fibroblast-derived IPSCs remained closer to parental fibroblasts. On denuded air-lifted corneas, limbal-derived IPSCs even upregulated differentiated corneal CK3 and CK12. Taking all the data together, the authors emphasize the importance of the natural niche and the limbal tissue of origin in generating IPSCs as LSCs for clinical aims [81]. Compared to the previous work of Hayashi and colleagues [80], this method presents two interesting improvements. The differentiation medium is serum free and contains defined growth supplements, allowing for a more standardized protocol and bringing it closer to a clinical application. Also, differentiating the IPSCs on hAM provides an advantage for the success of future transplantation [21]. Both research [80,81] lead to the conclusion that the initial cell type from which IPSCs are derived is important for the quality of the final LSC-like cells obtained. However, for clinical applications easily accessible donor cell types should be identified to create IPSCs-LSCs. Other than fibroblasts, adult progenitor cells like bone-marrow or hair follicle -derived mesenchymal stem cells should be also tested.

In an elegant approach applying a directed two-stage differentiation protocol without the use of feeder cells or serum in the culture medium, researchers generated relatively pure populations of corneal epithelial-like progenitor cells capable of terminal differentiation toward mature corneal epithelial-like cells [82]. Early developmental mechanisms could be reproduced *in vitro* by blocking the transforming growth factor β (TGF-β) and Wnt-signaling pathways with small molecule inhibitors and activating bFGF signaling. IPSCs were cultured onto collagen IV substrate in specific corneal epithelial cell growth media which differentiated them into LSCs. Cells expressed typical LSC markers such as cytokeratins (CK3, CK12 and CK15) as well as Pax6, ABCG2 and ΔNp63 after five weeks of differentiation [82]. Interestingly, the differentiation protocol described by the authors, using growth factors and small molecules inhibitors, can be performed totally in xeno-free, feeder-free and serum-free conditions, allowing for a reproducible and clinical grade production of the IPSCs-LSCs ready to be used in the clinical setting. To bring one more step closer into the clinic, Wu’s team [83] described a IPSCs-LSCs transplantation system that introduces a 3D scaffolding in which IPSCs are seeded, differentiated and grafted into an acellular porcine matrix scaffold. This bioengineering system is aimed to overcome the gradual loss of viability over time of LSC grafted cells and the limitations of amniotic membrane. The method improved the outcome in rabbit experimental models [83].

In conclusion, even if several adult stem cells types have been used for regeneration of corneal epithelium, LSCs themselves have shown superior results and are the cells of choice for LSCD treatment. Since IPSCs grow indefinitely, IPSC-derived LSCs are an unlimited source of autologous LSCs for patients with bilateral LSCD – and therefore no LSCs left - and to avoid the risks of surgical intervention in unilateral LSCD. Moreover, the idea of creating IPSCs banks to provide HLA matched (immune-compatible) tissues is being strongly considered by the scientific community [84]. This would provide a ready-to-go source of material for LSCs derivation avoiding the high cost of personalized IPSCs development.

### 3.2. Molecular Mechanisms of Corneal Epithelial Reprogramming

Molecular mechanisms of epithelial reprogramming have been analyzed, and ΔNp63 has emerged as a central protein in IPSC reprogramming routes. ΔNp63 has been found to enhance IPSCs generating efficiency, as the loss of function of this protein decreased the mesenchymal-epithelial transition (MET) and pluripotency genes [85]. Accordingly, in cell oncogenic transformation (a process that may share similarities with IPSCs reprogramming at signaling level) it was found that Oct4 upregulation could enhance the expression of ΔNp63 while repressing p53 [86]. APR-246/PRIMA-1^met^ (a small compound which restores the functionality of mutant p53 in human tumor cells that target mutant forms of ΔNp63) was found to reverse corneal epithelial lineage commitment and to reinstate a normal p63-related signaling pathway [87]. In this study [87], the authors designed a unique cellular model that recapitulated major embryonic defects related to ectrodactyly ectodermal dysplasia cleft (lip/palate) syndrome (EEC syndrome), which is caused by single point mutations in the *p63* gene. Fibroblasts from healthy donors and from EEC patients carrying two different point mutations in the DNA binding domain of p63 were reprogrammed into IPSC lines. Phenotypic defects in EEC syndrome include skin defects and LSCD, with loss of corneal transparency. In this interesting *in vitro* model, EEC-derived IPSCs failed to terminal differentiate into CK14 cells (epidermis and LSCs) or CK3/CK12 cells (corneal epithelial cells) [87]. This research team also described previously the possible roles of specific miRs in corneal development using IPSC corneal differentiation methods [88]. Similarly, IPSC epithelial somatic differentiation seems to recapitulate the molecular steps during embryonic development, in which ΔNp63 is a master regulator of epithelial differentiation. Moreover, during IPSCs generation it is widely accepted that MET is needed [89]. Blocking MET during cell reprogramming (using TGF-β or Snail1) prevents IPSCs induction. In this change of cell state, the inverse of MET occurs during embryonic development, in which epithelial-mesenchymal transition (EMT) pointing out the parallelism between embryonic development and cell reprogramming [89]. Another interesting signaling pathway that may be involved in the IPSC differentiation into epithelial cells is the Pax6/β-catenin pathway [90]. During the embryonic development of the chicken eye, eye specification seems to be established by the inhibition of the canonical Wnt pathway and TGF-β, which induces the upregulation of Pax6 in the lens ectoderm [87]. In support of this theory, the trans-differentiation of multipotent hair follicle stem cells into corneal epithelial-like cells is mediated by the upregulation of Pax6 and the inhibition of the canonical Wnt-signaling pathway [73]. Thus, further investigation is needed to clarify whether this mechanism really affects the differentiation of IPSCs into corneal epithelial cells.

### 3.3. Restoration of Corneal Stromal Transparency

The restoration of corneal transparency after stromal or endothelial damage is another field of interest in which IPSCs generation and differentiation may have an impact. The production of corneal keratocytes from pluripotent cells also has significant implications for cell-based therapy and tissue engineering for treatment of corneal diseases. At present, however, there are very few studies of the use of IPSCs as an effective and conclusive approach for cell therapy applications for recovery corneal stroma, and the results are very preliminary.

Funderburgh’s group [91] developed a methodology for inducing the differentiation of human embryonic stem cells (hESCs) into cells with a gene-expression phenotype similar to that of adult human corneal keratocytes. The transparency of the cornea depends on the unique molecular composition and organization of the extracellular matrix of the stroma (collagen fibrils), which is a product of keratocytes—specialized neural crest (NC)-derived mesenchymal cells. In Funderburgh’s study, neural differentiation of the hESC cell line was induced by co-culture with mouse PA6 fibroblasts as a feeder-layer. After a few days in co-culture, hESCs acquired the ability to express cell-surface nerve growth factor receptor (NGFR, p75NTR) of low affinity. These cells were then isolated from co-cultures by immunoaffinity adsorption and cultured further as a monolayer. Corneal keratocyte phenotype was induced in serum-free medium containing ascorbate and was independent of the substratum for cultivation. Interestingly, hESC co-cultures upregulated the expression of some specific *NC* genes, and when NGFR-expressing cells were expanded as a monolayer, mRNAs typifying adult stromal stem cells were detected. Further, when these cells were cultured as substratum-free pellets, several corneal keratocyte markers were upregulated, among them keratocan, a corneal stroma-specific proteoglycan. The analysis of culture medium obtained from the pellets also contained high concentrations of keratocan modified with keratan sulfate, considered a unique molecular component of corneal stroma. This study showed the possibility to differentiate keratocytes *in vitro*. The authors also hypothesized that IPSCs derived from adult somatic cells could be used in place of hESCs for both, to provide autologous material for bioengineered corneal matrix or for direct stromal cell-based therapy [91].

Human corneal keratocytes could also be reprogrammed into IPSCs exhibiting pluripotent properties. To prevent feeder cell contamination and to improve the clinical utility of reprogrammed IPSCs, Chien and colleagues [92] developed a feeder-free (without MEF, mouse embryonic fibroblasts cells) and serum-free method to stably expand human IPSCs *in vitro*. This approach allows cells to remain stable through 30 passages, maintaining ESC-like pluripotent properties. Furthermore, to improve IPSCs delivery and engraftment, a biocompatible injectable nanogel (thermo-gelling carboxymethyl-hexanoyl chitosan; CHC) was developed. The authors also evaluated whether the viability and pluripotent properties of human corneal keratocyte-derived IPSCs can be retained in a CHC hydrogel system, and explored the therapeutic potential of these cells on corneal impairment using CHC hydrogel as delivery vehicle in a rat model of corneal damage induced by either chemical burns or surgical ablation. They concluded that the IPSC/CHC system enhanced corneal regeneration by downregulating oxidative stress and recruiting endogenous epithelial cells to restore corneal epithelial thickness, and also reconstructing the corneal microenvironment niche [92].

Very recently, Fukuta and co-researchers [93] developed an efficient induction protocol using chemically defined culture medium containing inhibitors for TGF-β signaling and inhibitors for Wnt-signaling pathway (GSK3β). This approach allow differentiate human neural crest cells (hNCC) from human pluripotent cells, with the same efficiency (70%–80%), independent of the parental cell type (ESCs or IPSCs), or method of generation (viral-integrated or plasmid-episomal). Furthermore, cells have been kept under feeder-free and xeno-free culture systems. Interestingly, generated hNCCs could be differentiated into corneal endothelial cells, among other complex cell types, such as peripheral neurons, glial cells and melanocytes. Endothelial cells of the cornea have been differentiated culturing hNCCs in corneal endothelial cell conditioned medium supplemented with selective ROCK inhibitor (Y-27632). After two weeks of induction, cells changed their morphology into that of polygonal corneal endothelial-like cells and started to express ZO-1, type IV and type VIII collagens, which are recognized corneal endothelial cell markers [93]. These results also open new and promising perspectives for possible clinic applications in corneal pathologies where the endothelium is primarily affected.

### 3.4. Future Trends for IPSC Technology

The ideal source of cells for ocular clinical application needs to meet certain criteria: (a) easy accessibility and minimal risks for patients; (b) availability in sufficient quantities for bio-replacement; and (c) a high likelihood of successful reprogramming [72]. However, present evidence confirms that the methods involving IPSCs production should be considered with caution before immediate clinical application. An example is exome sequencing of several human IPSC lines, identified over a hundred point mutations in the generated cells but not in the parental cells. Many missense mutations associated with the function of different proteins and other point mutations in genes related to cancers have been observed [94]. In this sense, a better understanding of the molecular mechanisms for differentiation into various cell types associated with more directed protocols for the reprogramming, without the need to induce complete states of non-differentiation, could contribute to mitigate possible aberrations in the genome of produced cell populations.

## 4. Conclusions

In recent years there have been significant developments in the use of cell-based cultures combined with biomaterials or biocompatible substrates for corneal epithelial tissue engineering bio-replacement. Current approaches for improving these therapeutic strategies include standardization of culture conditions and development of xenobiotic free culture systems, evaluation of novel bio-functional scaffolds to enhance stem cell expansion and transplantation efficacy, and exploration of alternative autologous progenitor cell sources. To date, the search for innovative strategies and approaches in the field of ocular surface reconstruction has produced some encouraging results. Several new strategies have emerged for future therapies for LSCD, although the best cell source and the ideal technique still need to be established. One of the key elements is the role of the cellular microenvironment or niche. The limbal stem cell niche contains stem cells that promote proliferation and migration and have immunosuppressive mechanisms to protect them from immunological reactions. The current findings suggest that the CLET and COMET approaches using autologous epithelial progenitor cells are the most widely accepted clinical techniques for treating LSCD.

One emerging alternative cell source for treating the ocular surface is the use of adult stem cells, which provide high proliferative potential, differentiated capability and lower immunogenicity; they are non-tumorigenic and can be obtained by minimally invasive methodologies. They represent a more physiological, more rational, and less invasive treatment. Meanwhile, stem cells from adult tissue, as in the case of mesenchymal stem cells, although they have showed an intrinsic potential for a possible epithelial differentiation, this has not yet been achieved. Also, the prospects for therapies derived from autologous mesenchymal and IPSCs that may yield a multitude of engineered tissue types are exciting. Although IPSCs are yet to be used for ocular surface reconstruction, a recent study has shown successful corneal epithelial cell generation. The search for alternative sources of stem cells in the treatment of ocular surface diseases represents a challenge. IPSCs represent a very promising option for obtaining corneal epithelial cells to apply in cell-based therapy for the ocular surface. In the future, a deeper understanding of the behavioral characteristics of the LSC niche as well as of proliferation and differentiation pathway events should help to expand and develop the use of IPSCs in ocular surface regenerative medicine.
